# Flemish breast cancer screening programme: 15 years of key performance indicators (2002–2016)

**DOI:** 10.1186/s12885-019-6230-z

**Published:** 2019-10-28

**Authors:** M. Goossens, I. De Brabander, J. De Grève, C. Van Ongeval, P. Martens, E. Van Limbergen, E. Kellen

**Affiliations:** 1 Vrije Universiteit Brussel, Laarbeeklaan 103, 1090 Brussels, Belgium; 2 Centrum voor Kankeropsporing (Centre for Cancer Detection), Ruddershove 4, 8000 Brugge, Belgium; 3 Belgian Cancer Registry, Rue Royale 215, 1210 Brussels, Belgium; 4 University Hospital Leuven, Campus St. Rafael, Kapucijnenvoer 33, 3000 Leuven, Belgium

## Abstract

**Background:**

We examined 15 years of key performance indicators (KPIs) of the population-based mammography screening programme (PMSP) in Flanders, Belgium.

**Methods:**

Individual screening data were linked to the national cancer registry to obtain oncological follow-up. We benchmarked crude KPI results against KPI-targets set by the European guidelines and KPI results of other national screening programmes. Temporal trends were examined by plotting age-standardised KPIs against the year of screening and estimating the Average Annual Percentage Change (AAPC).

**Results:**

PMSP coverage increased significantly over the period of 15 years (+ 7.5% AAPC), but the increase fell to + 1.6% after invitation coverage was maximised. In 2016, PMSP coverage was at 50.0% and *opportunistic coverage* was at 14.1%, resulting in a total coverage by screening of 64.2%. The response to the invitations was 49.8% in 2016, without a trend. Recall rate decreased significantly (AAPC -1.5% & -5.0% in initial and subsequent regular screenings respectively) while cancer detection remained stable (AAPC 0.0%). The result was an increased positive predictive value (AAPC + 3.8%). Overall programme sensitivity was stable and was at 65.1% in 2014.

In initial screens of 2015, the proportion of DCIS, tumours stage II+, and node negative invasive cancers was 18.2, 31.2, and 61.6% respectively. In subsequent regular screens of 2015, those proportions were 14.0, 24.8, and 65.4% respectively. Trends were not significant.

**Conclusion:**

Besides a suboptimal attendance rate, most KPIs in the Flemish PMSP meet EU benchmark targets. Nonetheless, there are several priorities for further investigation such as a critical evaluation of strategies to increase screening participation, organising a biennial radiological review of interval cancers, analysing the effect that preceding opportunistic screening has on the KPI for initial screenings, and efforts to estimate the impact on breast cancer mortality.

## Introduction

Breast cancer (BC) is a leading cause of disease burden among women in Europe: an estimated 522,513 women were diagnosed with BC in 2018, and 137,707 died of BC that year (GLOBOCAN 2018). Mammographic screening can reduce BC mortality in women over 50 years old, although the magnitude of this mortality reduction is the subject of ongoing debate. Estimates range from 20% or less for the group invited to screening, to 48% for the group that gets screened [1, 2]. Mammographic screening also has limitations, including the occurrence of interval cancers and diagnosing BC that never would have been diagnosed nor caused symptoms in the absence of screening (overdiagnosis).

Many countries offer mammographic screening in the framework of a population-based mammography screening programme (PMSP), which aims to give all asymptomatic women in the target population systematic and equal access to screening while quality assurance and data collection are performed in a centralized manner. A PMSP can exist in parallel with opportunistic screening, which follows the spontaneous initiative of the woman or her physician [3].

Using breast cancer mortality as an endpoint in the evaluation of a PMSP seems obvious, but it takes many years before an effect on mortality can be observed [4]. Key performance indicators (KPIs) cannot replace a mortality analysis, but enable programmes to compare performance against objectives. Monitoring and evaluating KPIs (such as cancer detection rate or programme sensitivity) is a necessity for public health interventions such as a PMSP to justify the use of public means [1, 4].

We calculated KPIs for the Flemish PMSP for the years 2002–2016, benchmarked crude KPI results against KPI-targets set by the European guidelines and KPI results of other national screening programmes, and examined temporal trends in age-standardised KPIs.

## Methods

### General outline of the PMSP in Flanders

Flanders is the most populated region in Belgium and has had a PMSP since June 2001. The Flemish PMSP is organized, coordinated, and monitored by the Centre for Cancer Detection (CvKO), in close collaboration with the Belgian Cancer Registry (BCR). Women aged 50–69 can have a screening every other calendar year, consisting of a two-view mammogram of both breasts without ultrasound or clinical breast examination. The screenings can be performed in 161 certified mammogram units and are paid directly and entirely by the Belgian healthcare insurance companies to the accredited mammogram units. Screening with digital mammography started in May 2007 and in 2016 99% of the screening exams were digital. Digital Radiography (DR) accounts for about two-thirds of all digital equipment.

The mammograms are read independently by two certified screening radiologists. Both readers categorize mammograms according to a five-category classification similar to BI-RADS (Breast Imaging-Reporting and Data System) [5]. Classes III (probably benign), IV (suspicious abnormality), and V (highly suspicious lesion) are recalled for diagnostic assessment. If the two readers do not reach the same conclusion, a third radiologist performs the third (and decisive) reading.

All results are sent to women (by post) and their physicians (electronically, and also by post in case of a suspicious finding). The physician’s letter describes breast density, type of lesion, location of the lesion, and advice regarding the nature of diagnostic assessment, and it is sent 3 days before the woman’s letter. Diagnostic assessment can take place in any radiological centre.

### Two pathways of PMSP participation

There are two pathways by which a woman can get screened in the PMSP. In pathway-1-screenings, physicians specifically prescribe a PMSP screening. This prescription is equal to a PMSP letter of invitation as in pathway − 2-screenings (see below). Pathway-1-screenings are reported as self-registration since these women did not receive an invitation prior to their participation. This pathway is not a safety net for unequal access to the PMSP, but rather meant to acknowledge the fact that some physicians have an excellent physician-patient relationship, rendering an invitation unnecessary. Women can be screened on a regular basis in pathway 1 for many years, without ever receiving an invitation.

In pathway 2, the CvKO uses the list of the eligible population to send out invitations by post every 2 years (eligible population is explained in the next section). Invitations contain an appointment to a certified mammogram unit, which can be altered by calling a toll free number. Besides this letter, there is no other formal system to remind women of an upcoming appointment.

### Population

The *target* population includes all women in Flanders aged 50–69, identified with the central population registry.

The *eligible* population excludes from the target population all women who had a bilateral mastectomy or BC in the last 10 years, by using a unique 11-digit personal identification number to cross-link each individual of the target population to the BCR. This exclusion is performed twice per year, before sending out the invitations that are scheduled to be sent out over the following 6 months.

All women from the eligible population should receive an invitation the same year, except women who:

- actively opted out;
- already had a PMSP screening in the previous year;
- were already invited in the previous year;
- had a pathway-1-screening in the current year.

We calculate **invitation coverage** to assess whether all these women did indeed receive an invitation.

### Opportunistic screening in Flanders

Women can also have a mammogram outside the PMSP. These mammograms are billed to the health insurance as “diagnostic mammograms”, they follow the spontaneous initiative of the woman or her physician, and require a prescription that is different from the prescription that is used for a Pathway-1-screening. The results of these mammograms are communicated at the end of the exam and there is no systematic second reading. These mammograms can either have a diagnostic indication (women with symptoms of breast cancer or meant as diagnostic assessment) or be intended for opportunistic screening (women without symptoms of breast cancer). Because data on diagnostic mammograms are not stored centrally, the total number of these mammograms can only be obtained with reimbursement records. Unfortunately, reimbursement records cannot distinguish between mammograms performed for a diagnostic indication and those done for opportunistic screening.

We therefore consider all of these mammograms as opportunistic screening, even though some of them were undoubtedly for diagnostic purposes (see below, Determining screening status).

### Oncological follow-up of screenings

The BCR collects data concerning all new cancer cases in Belgium and has access to health insurance reimbursement data. The completeness of the BCR breast cancer data was previously estimated to be 99.7% [6]. At the time of screening, women are given the possibility to opt-out of their data being used for research. Refusal rates fluctuate around 1% or less of screened women. The national privacy commission approved using a unique 11-digit personal identification number to cross-link each consenting screened individual to the oncological data from the BCR. Relevant BCR data can therefore be used as oncological follow-up for every consenting screened woman. This is currently the only source of follow-up data.

### Determining screening status

We report on two types of participation data:

- **Invitation response**
  - Percentage of women who got a *PMSP screening* within 24 months after receiving their invitation (The invitation is valid up to 24 months after being sent).
- **Coverage**
  - The basis of our coverage data was the eligible population. Since the eligible population fluctuates throughout the year (death, immigration, etc.), we used the data of the first of January of each year as the basis for coverage data. The Flemish Working Group on breast cancer screening developed a method to determine coverage status for all of these women: check for opportunistic screening and PMSP screening in year x and x-1 and then use Table 1 to categorise. Data on opportunistic screening coverage cannot be reliably calculated for 2002.

**Table 1:** Determining coverage status in year x

Screening year x-1	Screening year x	Coverage year x
No screening	No screening	No coverage
	PMSP	PMSP coverage
	Opportunistic	Opportunistic coverage
	PMSP & Opportunistic	PMSP coverage
Opportunistic	No screening	Opportunistic coverage
	PMSP	
	Opportunistic	
	PMSP & Opportunistic	
PMSP	No screening	PMSP coverage
	Opportunistic	
Opportunistic & PMSP	No screening	Most recent mx in year x-1 determines coverage type
	Opportunistic	

### Definitions

The definitions in Table 2 were used together with the above descriptions of population and screening status.

**Table 2:** Definitions used

Breast cancer	A first diagnosis of invasive carcinoma or *ductal* in-situ carcinoma of the breast (respectively C50 and D05 of ICD-O, third edition, version 10).
Cancer detection rate	The number of breast cancers detected in a screening round per 1000 women screened.
False-positive recall	Any recall for diagnostic assessment that was not followed by a screen-detected cancer.
False-positive recall rate	The number of women with a False-positive recall per 1000 women screened.
Initial screening	The first screening examination of individual women within the PMSP, regardless of how long the programme has been running
Interval cancer	• Breast cancer that was diagnosed within 24 months of a negative screen. • Breast cancer that was diagnosed more than 3 months after the first diagnostic assessment that followed a positive screen (but at the latest within 24 months of screening).
Interval cancer rate	The number of interval cancers diagnosed per 1000 women screened.
Invitation coverage	The number of women that receive an invitation in year x, as a proportion of all women that should be invited in that year.
Positive predictive value	The number of breast cancers detected per 100 women recalled for diagnostic assessment.
Programme sensitivity	The number of screen-detected cancers as a proportion of all breast cancers discovered in the screened population within 2 years of screening
Proportion of node-negative cancers	The number of node-negative cancers as a proportion of the total number of invasive screen-detected cancers
Proportion of DCIS	The number of DCIS as a proportion of the total number of screen-detected cancers
Proportion of stage ≥2	The number of Stage II+ breast cancers as a proportion of the total number of screen-detected cancers
Recall rate	The number of women recalled for diagnostic assessment per 100 women screened.
Screen-detected cancer	Breast cancer that was diagnosed within 3 months of the first diagnostic assessment that followed a positive screen (but at the latest within 24 months of screening).
Subsequent irregular screening	Any screening examination after the initial screening, where the most recent PMSP screening occurred > 30 months after the previous PMSP screening
Subsequent regular screening	Any screening examination after the initial screening, where the most recent PMSP screening occurred <=30 months after the previous PMSP screening

### Statistical analysis

We included all screening mammograms made for women 50–69 years old during the period 2002–2016. Crude KPIs were calculated as described above, stratified by year of screening, and reported separately for initial and subsequent screenings (see Table 2). Age-standardised KPIs were calculated using the world standard population [7].

We benchmarked our crude KPI results against KPI results of other national screening programmes, and the KPI-targets set by the European guidelines for quality assurance in breast cancer screening [4].

Age-standardised KPIs were plotted against the year of screening to analyse temporal trends. APCs (Annual Percentage Change) were estimated from least squares regressions on the logarithm of the age-standardised KPIs versus year of screening. APC is to be interpreted as the mean multiplicative change per year (relative percentage change). If a trend could not be considered linear over the entire interval (on a log scale), the Average Annual Percentage Change (AAPC) was calculated instead of the APC. The AAPC is calculated as the average of the APC estimates of several segments, weighted by the corresponding segment length. In each of these segments the trend (on a log scale) can be considered linear [8]. This method has been used in many studies in a variety of fields to identify temporal patterns [9, 10].

We used the Joinpoint Regression Programme (version 4.7.0) developed by the US National Cancer Institute, to estimate the models that best fitted the data (default setting, Permutation Test) and to calculate AAPC. When a KPI had several joinpoints, we also report the APC of the last segment, since this can give interesting information about the most recent trend. All other analyses were conducted using Stata version 13 (StataCorp., USA); significance was set at *p* < 0.05.

## Results

Table 1 shows that between 2002 and 2016, a total of 2,613,737 PMSP screenings were performed, of which a BCR link was established for 97.7%. These women had a mean age of 58.6 (years).

### Participation

**Fig. 1:**
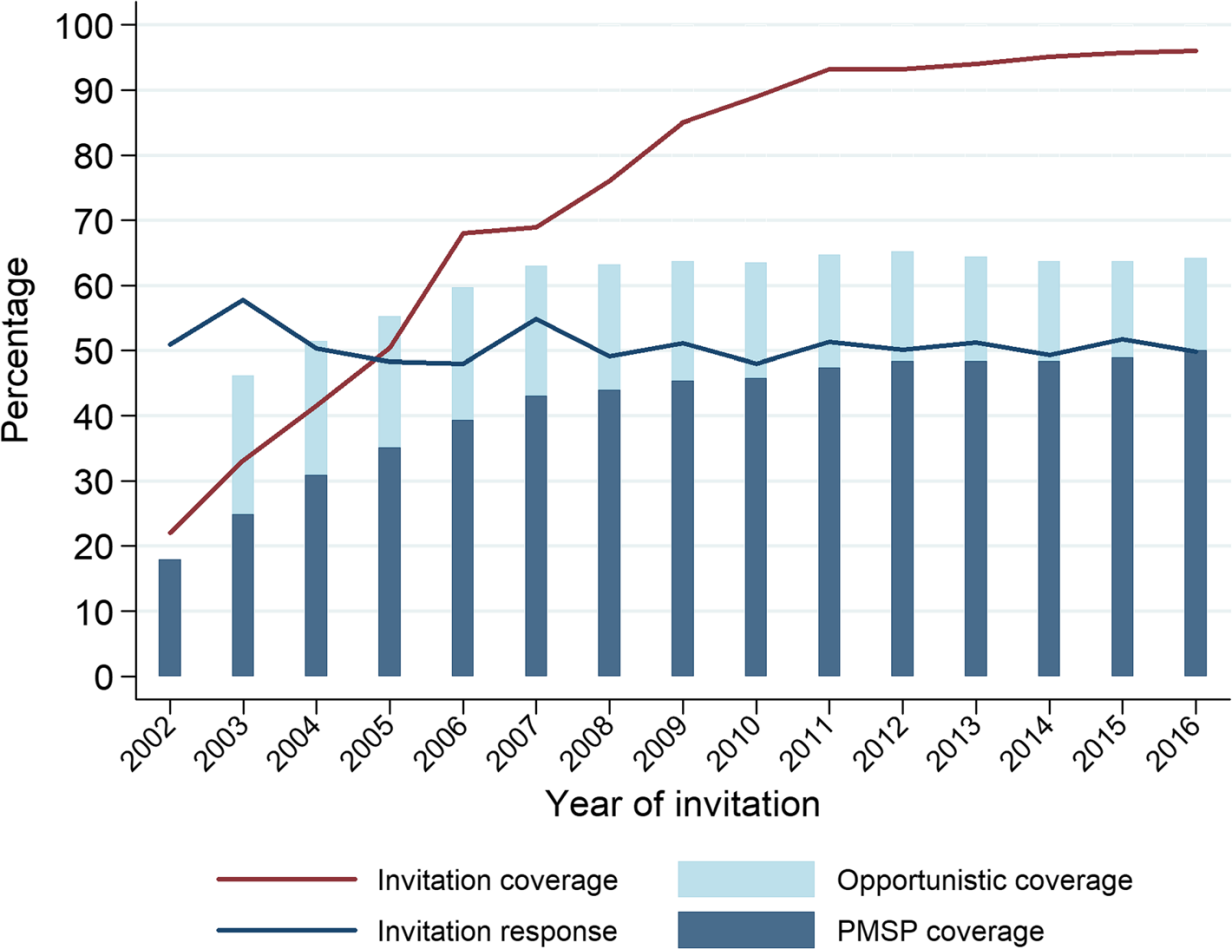
Invitation coverage, invitation response and different types of coverage, Flanders Belgium 2002–2016

In the first 10 years of the PMSP, the proportion of women receiving an invitation was suboptimal: invitation coverage did not reach 90% until 2011 and achieved 96.0% in 2016 (see Fig. 1 and Table 3). PMSP coverage was at 50.0% in 2016 and *opportunistic coverage* was at 14.1%, resulting in total coverage by screening at 64.2%. PMSP coverage increased significantly (+ 7.5% AAPC), but the increase mainly occurred between 2002 and 2007 (APC + 14.2%), coinciding with the sharp rise in invitation coverage. After 2007, the AAPC is still positive but falls to + 1.6%. The response to the invitations was 49.8% in 2016 and did not display an upwards trend since the initiation of the programme.

**Table 3:** Key performance indicators (invitation, participation, recall and cancer detection) of the Population-based Mammographic Screening Programme, Flanders Belgium 2002–2016

Performance indicator [EU desirable target]	2002	2003	2004	2005	2006	2007	2008	2009	2010	2011	2012	2013	2014	2015	2016	AAPC all years		APC^b^ last segment		
																%	(95% CI)	Year^a^	%	(95% CI)
Target population 01/01, N	**692,964**	**699,472**	**707,645**	**717,956**	**728,628**	**739,449**	**751,171**	**763,832**	**778,866**	**799,767**	**816,921**	**830,455**	**843,110**	**854,612**	**861,223**					
BC or Bilateral mastectomy, N	**13,569**	**15,886**	**18,248**	**19,989**	**20,833**	**21,689**	**22,522**	**22,985**	**23,400**	**23,935**	**24,680**	**25,220**	**25,810**	**26,226**	**26,474**					
Eligible - refuses to be invited, N	**12,364**	**13,693**	**14,998**	**16,264**	**17,481**	**18,702**	**19,757**	**21,001**	**21,991**	**23,089**	**23,863**	**24,625**	**24,962**	**25,223**	**24,809**					
Eligible - not to be invited this year^b^, N	**45,207**	**171,528**	**198,729**	**227,791**	**255,135**	**314,695**	**279,757**	**333,516**	**335,365**	**360,949**	**371,003**	**370,777**	**380,358**	**386,578**	**390,457**					
Eligible - to be invited this year, N	**621,824**	**498,365**	**475,670**	**453,912**	**435,179**	**384,363**	**429,135**	**386,330**	**398,110**	**391,794**	**397,375**	**409,833**	**411,980**	**416,585**	**419,483**					
Invitations sent (invitation coverage), %	22.0	33.1	41.5	50.4	68.0	68.9	76.1	85.0	88.9	93.2	93.1	94.0	95.1	95.7	96.0	+ 10.5^b^	(+ 8.3; + 12.8)	2011	+ 0.4	(−2.8; + 3.8)
Eligible population 01/01, N	**679,395**	**683,586**	**689,397**	**697,967**	**707,795**	**717,760**	**728,649**	**740,847**	**755,466**	**775,832**	**792,241**	**805,235**	**817,300**	**828,386**	**834,749**					
PMSP coverage, %	17.9	24.8	30.9	35.0	39.4	43.1	44.0	45.4	45.8	47.4	48.4	48.4	48.3	48.9	50.0	+ 7.5^b^	(+ 6.5; + 8.6)	2007	+ 1.6^b^	(+ 1.1; + 2.0)
Opportunistic coverage, %	unknown	21.3	20.5	20.2	20.3	19.9	19.2	18.3	17.7	17.2	16.8	16.0	15.3	14.8	14.1	−3.0^b^	(−3.3; −2.6)	2007	−3.6^b^	(−3.3; −2.6)
No coverage, %		53.8	48.6	44.7	40.4	37.0	36.8	36.4	36.6	35.3	34.8	35.6	36.3	36.3	35.8	−3.0^b^	(−3.5; −2.5)	2012	+ 0.7	(−0.4; + 1.8)
All invitations sent, N	**120,517**	**164,296**	**202,155**	**241,076**	**311,940**	**276,511**	**339,264**	**343,924**	**370,439**	**382,475**	**385,966**	**402,569**	**407,864**	**415,304**	**417,493**					
Invitation Response Rate, %	50.9	57.8	50.3	48.3	48.0	54.8	49.1	51.1	48.0	51.3	50.1	51.2	49.3	51.7	49.8	−0.3	(−0.9; + 0.4)			
All PMSP examinations, N	**103,909**	**119,861**	**127,714**	**134,772**	**164,075**	**165,873**	**176,133**	**183,175**	**183,810**	**203,326**	**199,899**	**209,809**	**204,540**	**218,723**	**218,118**					
With BCR link, %	80.0	83.3	98.7	98.9	98.6	98.9	98.7	98.9	99.0	99.4	99.4	99.5	99.6	99.7	99.8					
PMSP examinations with BCR link, N	**83,100**	**99,787**	**126,007**	**133,265**	**161,777**	**164,129**	**173,768**	**181,082**	**181,968**	**202,101**	**198,747**	**208,839**	**203,735**	**218,155**	**217,614**					
mean age, years	59.8	58.6	58.8	58.9	58.4	58.7	58.4	58.5	58.5	58.6	58.6	58.7	58.6	58.8	58.8					
initial screening, N	**83,100**	**89,385**	**62,149**	**46,728**	**55,899**	**44,823**	**40,562**	**42,923**	**38,995**	**40,506**	**40,961**	**36,852**	**37,497**	**38,454**	**38,851**					
mean age, years	59.8	58.4	56.2	57.1	55	54.7	54.2	53.5	53.5	53.2	53	52.7	52.7	52.6	52.8					
subsequent irregular screening, N		**12**	**16,138**	**15,935**	**21,479**	**19,179**	**15,886**	**16,714**	**17,911**	**18,950**	**17,676**	**17,207**	**16,863**	**19,490**	**21,045**					
mean age, years		58.9	61.2	60.3	60.3	60.5	60.6	60.7	60.3	60.6	60.8	60.7	60.7	60.8	60.8					
subsequent regular screening, N		**10,390**	**47,720**	**70,602**	**84,399**	**100,127**	**117,320**	**121,445**	**125,062**	**142,645**	**140,110**	**154,780**	**149,375**	**160,211**	**157,718**					
mean age, years		60.1	61.2	59.8	60.2	60.2	59.6	59.9	59.8	59.8	60	60	59.9	60	60					
Digital screening, %	0.0	0.0	0.1	0.3	1.9	9.0	21.4	45.6	59.6	79.5	88.7	95.3	95.2	96.8	98.9					
Women recalled for diagnostic assessment, N	**4377**	**5598**	**5774**	**5859**	**7488**	**6322**	**7567**	**6706**	**6038**	**5932**	**6066**	**5328**	**5232**	**5183**	**5585**					
Recall Rate, %	5.3	5.6	4.6	4.4	4.6	3.9	4.4	3.7	3.3	2.9	3.1	2.6	2.6	2.4	2.6					
initial screens, % [< 5%]	5.3	5.8	6	6.1	6.8	6.4	7.5	6.4	5.5	5.3	5.4	4.4	4.5	4.5	4.7	−1.5^b^	(−2.9; −0.1)	2008	**−5.1** ^b^	(−6.9; −3.3)
subsequent irregular screens, %			3.6	4.6	4.5	4.1	4.9	4.2	3.8	3.1	3.8	3.2	3.4	3.2	3.4	−2.9^b^	(−4.6; −1.1)			
subsequent regular screens, % [< 3%]		3.6	3.1	3.2	3.2	2.7	3.2	2.7	2.6	2.2	2.3	2	2	1.8	1.9	−5.0^b^	(−6.0; − 3.9)			
Screen detected cancers, N	**797**	**894**	**789**	**856**	**967**	**888**	**952**	**976**	**1018**	**1126**	**1153**	**1227**	**1123**	**1195**	**1190**					
Cancer Detection Rate, ‰	9.6	9.0	6.3	6.4	6.0	5.4	5.5	5.4	5.6	5.6	5.8	5.9	5.5	5.5	5.5					
initial screens, ‰	9.6	9.2	7.2	8.0	7.4	6.6	7.1	6.5	6.3	6.5	6.3	5.8	5.6	6.4	6.3	−0.6	(−1.4; + 0.2)			
subsequent irregular screens, ‰			6.7	7.1	6.4	6.0	7.4	8.1	6.9	7.3	9.1	8.2	8.2	9.0	7.5	+ 2.1^b^	(+ 0.3; + 3.9)			
subsequent regular screens, ‰		6.4	4.9	5.2	4.9	4.8	4.7	4.6	5.2	5.1	5.2	5.6	5.2	4.8	5.0	0.0	(−1.1; + 1.1)			
False-Positive Recalls, N	**3582**	**4704**	**4986**	**5003**	**6521**	**5434**	**6615**	**5730**	**5020**	**4806**	**4913**	**4101**	**4109**	**3988**	**4395**					
False-Positive Recall Rate, ‰	43.1	47.1	39.6	37.5	40.3	33.1	38.1	31.6	27.6	23.8	24.7	19.6	20.2	18.3	20.2					
initial screens, ‰	43.1	49.1	52.8	53.4	60.4	57.7	67.7	57.9	49.1	46.2	47.7	38.5	39.7	38.1	40.3	−1.4	(−3.5; + 0.8)	2007	**−5.3** ^b^	(−7.4; − 3.1)
subsequent irregular screens, ‰			29.6	39.3	38.6	34.9	41.5	33.8	31.2	24.0	29.0	23.9	25.7	22.6	26.2	−4.0^b^	(−6.1; −1.9)			
subsequent regular screens, ‰		29.2	25.7	26.6	27.4	21.8	27.4	22.1	20.4	17.4	17.5	14.7	14.6	13.0	14.4	−6.1^b^	(−7.4; −4.9)			
Positive predictive value, %	18.2	16.0	13.6	14.6	12.9	14.0	12.6	14.6	16.9	19.0	19.0	23.0	21.5	23.1	21.3					
initial screens, %	18.2	15.8	12.0	13.0	10.9	10.2	9.5	10.2	11.3	12.3	11.7	13.1	12.4	14.4	13.6	+ 0.1	(−2.5; + 2.8)	2006	**+ 3.8** ^b^	(+ 1.5; + 6.1)
subsequent irregular screening, %			18.4	15.3	14.2	14.8	15.2	19.4	18.2	23.3	23.9	25.5	24.3	28.4	22.3	+ 5.0^b^	(+ 2.5; + 7.4)			
Subsequent regular screening, %		17.9	15.9	16.5	15.2	18.0	14.6	17.3	20.3	22.6	23.0	27.8	26.2	27.1	25.7	+ 3.8^b^	(+ 0.6; + 7.0)	2013	−2.1	(−11.5; + 8.4)

Numbers in bold are absolute numbers

*AAPC* Average Annual Percentage Change, *BC* Breast Cancer, *PMSP* Population-based Mammographic Screening Programme, *BCR* Belgian Cancer Registry; (A)APC of participation data are calculated on non-age-standardised data

^a^the year of the last joinpoint is the beginning of the last segment

^b^indicates the (A)APC is significantly different from zero at the alpha = 0.05 level

### Recall rate & cancer detection

**Fig. 2:**
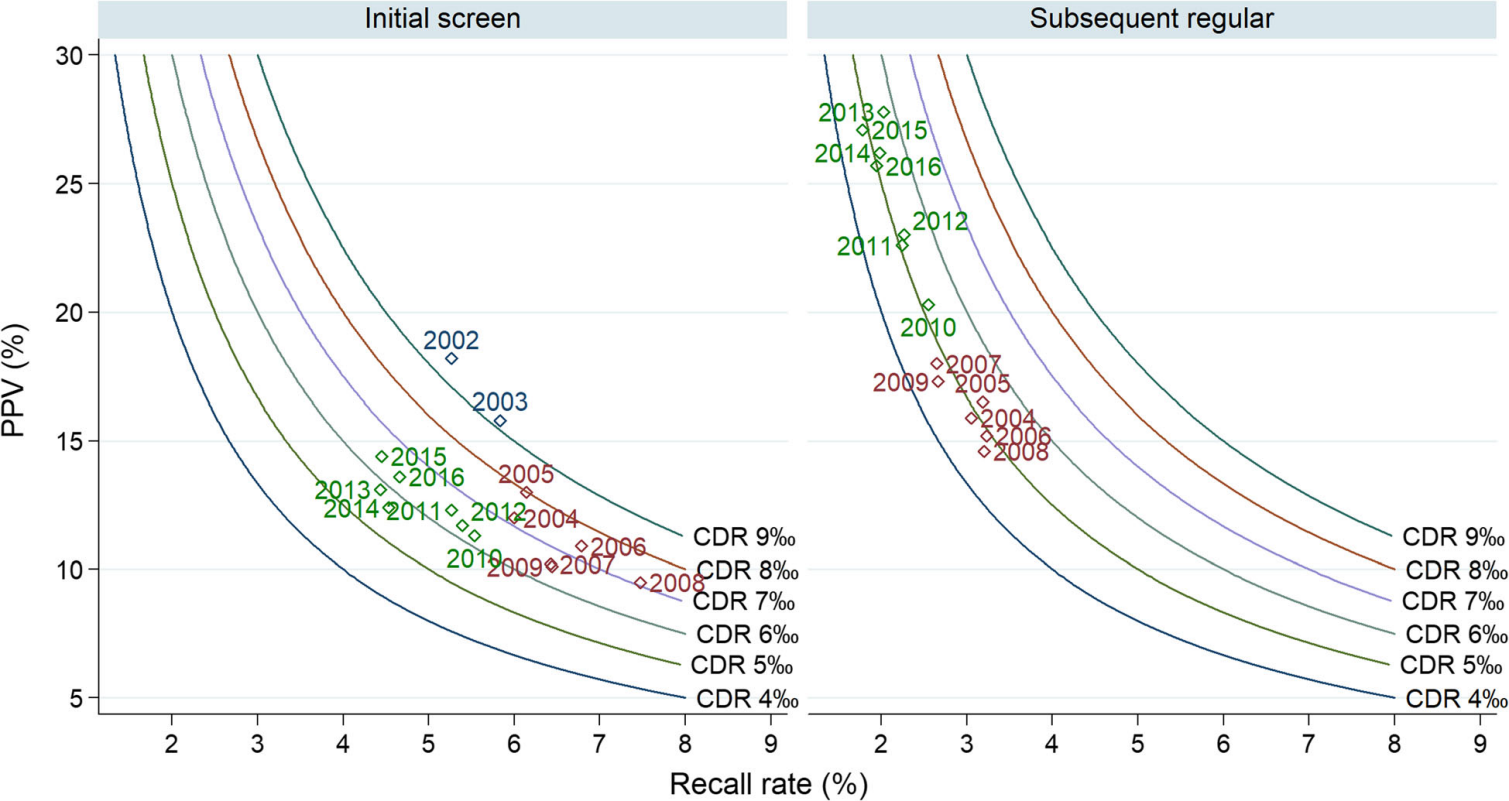
Recall rate versus positive predictive value, cancer detection rate shown as isobars. Analysed by screening round, Flanders Belgium 2002–2016

Figure 2 combines recall rates, positive predictive values, and cancer detection rates (as proposed by Blanks [11]). Figure 2 and Table 3 show that recall rate has decreased in initial and subsequent screenings (AAPC -1.5% & -5.0% in initial and subsequent regular screenings). In the subsequent regular screens a decrease in recall rates occurred together with a stable CDR (AAPC 0.0%), resulting in an increased positive predictive value (PPV) (AAPC + 3.8%).

### Interval cancers and sensitivity

Table 4 shows that overall programme sensitivity is stable and was 65.1% in 2014. There is only a significant trend in the initial screens (AAPC − 1.3%). Most of the interval cancers (62.9% for women screened in 2014) arise in the second year after screening (no significant trend). The majority of interval cancers appear after a *negative* screening. Nonetheless, 9.6% of all interval cancers occurring after a 2014 screening were found after a *positive* screening followed by a false negative diagnostic assessment. This proportion shows a clear decreasing trend (AAPC − 6.4%).

**Table 4:** Programme sensitivity, interval cancers and Screen detected cancer characteristics of the Population-based Mammographic Screening Programme, Flanders Belgium 2002–2016

Performance indicator [EU desirable target]		2002	2003	2004	2005	2006	2007	2008	2009	2010	2011	2012	2013	2014	2015	AAPC all years		APC last segment		
																%	(95% CI)	Year^a^	%	(95% CI)
All cancers (screen detected & interval cancers), N		**1071**	**1229**	**1147**	**1215**	**1407**	**1317**	**1450**	**1486**	**1543**	**1732**	**1702**	**1848**	**1726**	n.a.					
Screen detected cancers, N		**797**	**894**	**789**	**856**	**967**	**888**	**952**	**976**	**1018**	**1126**	**1153**	**1227**	**1123**	**1195**					
Programme sensitivity, %		74.4	72.7	68.8	70.5	68.7	67.4	65.7	65.7	66.0	65.0	67.7	66.4	65.1						
initial screens, %		74.4	73.0	69.4	71.2	72.3	66.4	65.8	68.5	65.5	67.7	70.6	61.7	61.0		−1.3^b^	(−2.4; −0.1)	2012	−6.5	(− 13.3; + 0.8)
subsequent irregular screens, %				73.0	72.0	70.3	69.0	71.1	73.9	67.0	66.7	71.2	73.8	68.8		−0.1	(−1.5; + 1.3)			
subsequent regular screens, %			68.8	65.9	69.3	65.0	67.7	64.5	62.7	66.0	63.8	66.1	66.6	65.6		+ 1.0	(− 0.5; + 2.5)	2005	− 0.4	(−1.2; + 0.5)
Interval cancers, N		**274**	**335**	**358**	**359**	**440**	**429**	**498**	**510**	**525**	**606**	**549**	**621**	**603**	n.a.					
Diagnosed in first year after screening, %		36.9	43.3	38.5	35.7	35.5	33.8	36.5	35.5	34.1	36.3	39.3	39.5	37.1		−0.1	(−1.3; + 1.1)			
Diagnosed after positive screen, %		22.3	21.5	17.0	12.3	16.6	14.0	16.3	12.9	10.3	11.7	10.0	10.5	9.6		−6.4^b^	(−8.5; −4.2)			
Interval cancer rate, ‰		3.3	3.4	2.8	2.7	2.7	2.6	2.9	2.8	2.9	3.0	2.8	3.0	3.0						
initial screens, ‰		3.3	3.4	3.2	3.2	2.8	3.3	3.7	3.0	3.3	3.1	2.6	3.6	3.6		+ 1.5	(−0.3; + 3.4)			
subsequent irregular screens, ‰				2.5	2.8	2.7	2.7	3.0	2.9	3.4	3.6	3.7	2.9	3.7		+ 3.2^b^	(+ 0.3; + 6.1)			
subsequent regular screens, ‰			2.9	2.5	2.3	2.6	2.3	2.6	2.7	2.7	2.9	2.7	2.8	2.7		+ 0.5	(−0.9; + 1.9)			
Characteristics screen detected cancers, initial screens																				
SDC total, N		**797**	**825**	**449**	**373**	**415**	**295**	**287**	**281**	**245**	**262**	**259**	**214**	**210**	**247**					
ductal carcinoma in-situ (DCIS), N		**140**	**132**	**72**	**59**	**75**	**50**	**58**	**53**	**41**	**64**	**35**	**48**	**35**	**44**					
invasive, N		**657**	**693**	**377**	**314**	**340**	**245**	**229**	**228**	**204**	**198**	**224**	**166**	**175**	**203**					
Stage, %	DCIS [10–20%]	17.6	16.0	16.0	15.8	18.1	16.9	20.2	18.9	16.7	24.4	13.5	22.4	16.7	18.2	−1.2	(−3.2; + 0.9)			
	Invasive SDC stage I	40.7	45.0	46.3	48.8	47.0	46.4	42.2	44.1	48.2	42.7	48.6	52.3	43.8	42.9					
	Invasive SDC stage ≥II [< 30%]	24.7	31.0	33.0	30.8	32.3	32.9	36.6	34.9	31.8	31.7	35.9	25.2	38.6	31.2	+ 1.9^b^	(+ 0.3; + 3.6)			
	Invasive SDC stage unknown	17.1	8.0	4.7	4.6	2.7	3.7	1.0	2.1	3.3	1.1	1.9	0.0	1.0	7.7					
Nodal status, %	Node - [> 70%]	51.0	61.5	62.9	66.2	62.6	65.3	62.9	67.5	63.7	68.7	64.7	71.7	63.4	61.6	+ 1.7	(−0.3; + 3.7)	2004	+ 0.1	(−0.8; + 1.1)
	Node +	20.2	23.5	26.3	22.3	26.8	25.7	32.8	29.4	29.4	28.3	29.0	25.9	30.9	24.6					
	Unknown (invasive SDC)	28.8	15.0	10.9	11.5	10.6	9.0	4.4	3.1	6.9	3.0	6.3	2.4	5.7	13.8					
Characteristics screen detected cancers, subsequent irregular screens																				
SDC total, N			**3**	**108**	**113**	**137**	**116**	**118**	**136**	**124**	**138**	**161**	**141**	**139**	**175**					
ductal carcinoma in-situ (DCIS), N			**0**	**15**	**11**	**15**	**21**	**17**	**21**	**21**	**23**	**28**	**19**	**20**	**25**					
invasive, N			**3**	**93**	**102**	**122**	**95**	**101**	**115**	**103**	**115**	**133**	**122**	**119**	**150**					
Stage, %	DCIS			13.9	9.7	10.9	18.1	14.4	15.4	16.9	16.7	17.4	13.5	14.4	14.9	+ 3.5	(−1.1; + 8.4)			
	Invasive SDC stage I			56.5	62.8	51.8	46.6	47.5	53.7	46.0	49.3	54.7	61.7	54.0	56.6					
	Invasive SDC stage ≥II			25.0	23.9	35.8	31.9	37.3	29.4	33.1	33.3	28.0	24.1	31.7	24.6	+ 2.6	(−4.5; + 10.2)	2007	−5.1	(−10.6; + 0.8)
	Invasive SDC stage unknown			4.6	3.5	1.5	3.4	0.8	1.5	4.0	0.7	0.0	0.7	0.0	4.0					
Nodal status, %	Node (−)			68.8	73.5	63.9	65.3	69.3	72.2	61.2	70.4	72.2	77.0	70.6	66.0	−0.0	(−1.6; + 1.6)			
	Node (+)			21.5	17.6	27.0	26.3	28.7	22.6	32.0	26.1	24.8	18.9	26.9	22.0					
	Unknown (invasive SDC)			9.7	8.8	9.0	8.4	2.0	5.2	6.8	3.5	3.0	4.1	2.5	12.0					
Characteristics screen detected cancers, subsequent regular screens																				
SDC total, N			**66**	**232**	**370**	**415**	**477**	**547**	**559**	**649**	**726**	**733**	**872**	**774**	**773**					
ductal carcinoma in-situ (DCIS), N			**10**	**36**	**70**	**78**	**83**	**95**	**91**	**93**	**106**	**110**	**141**	**120**	**106**					
invasive, N			**56**	**196**	**300**	**337**	**394**	**452**	**468**	**556**	**620**	**623**	**731**	**654**	**667**					
Stage, %	DCIS [10–20%]		15.2	15.5	18.9	18.8	17.4	17.4	16.5	14.5	14.6	15.0	16.2	16.1	14.0	+ 2.5	(−1.6; + 6.8)			
	Invasive SDC stage I		60.6	52.2	45.7	52.8	52.8	52.1	51.9	56.5	56.5	54.4	56.9	56.7	55.2					
	Invasive SDC stage ≥II [< 25%]		21.2	30.2	31.9	24.3	27.0	29.3	30.2	27.4	27.8	29.9	26.1	26.6	24.8	−0.9	(−2.2; + 0.5)			
	Invasive SDC stage unknown		3.0	2.2	3.5	4.1	2.7	1.3	1.4	1.5	1.1	0.7	0.8	0.5	6.0					
Nodal status, %	Node (−) [> 75%]		76.8	63.8	64.0	65.6	71.6	71.5	71.4	71.9	73.5	72.7	74.8	74.8	65.4	+ 0.1	(−1.3; + 1.5)			
	Node (+)		16.1	25.5	25.7	22.0	22.6	23.2	25.2	23.9	23.2	24.2	22.4	21.7	23.4					
	Unknown (invasive SDC)		7.1	10.7	10.3	12.5	5.8	5.3	3.4	4.1	3.2	3.0	2.7	3.5	11.2					

Numbers in bold are absolute numbers; N.A.: these data exist but are not yet available

*AAPC* Average Annual Percentage Change, *SDC* Screen Detected Cancer, *DCIS* Ductal carcinoma in-situ

^a^the year of the last joinpoint is the beginning of the last segment

^b^indicates the (A)APC is significantly different from zero at the alpha = 0.05 level

The interval cancer rate for screenings from 2014 on was 3.6/1000 and 2.7/1000 (initial and subsequent regular screens respectively), without a significant trend.

### Tumour stage of screen-detected cancers

**Fig. 3:**
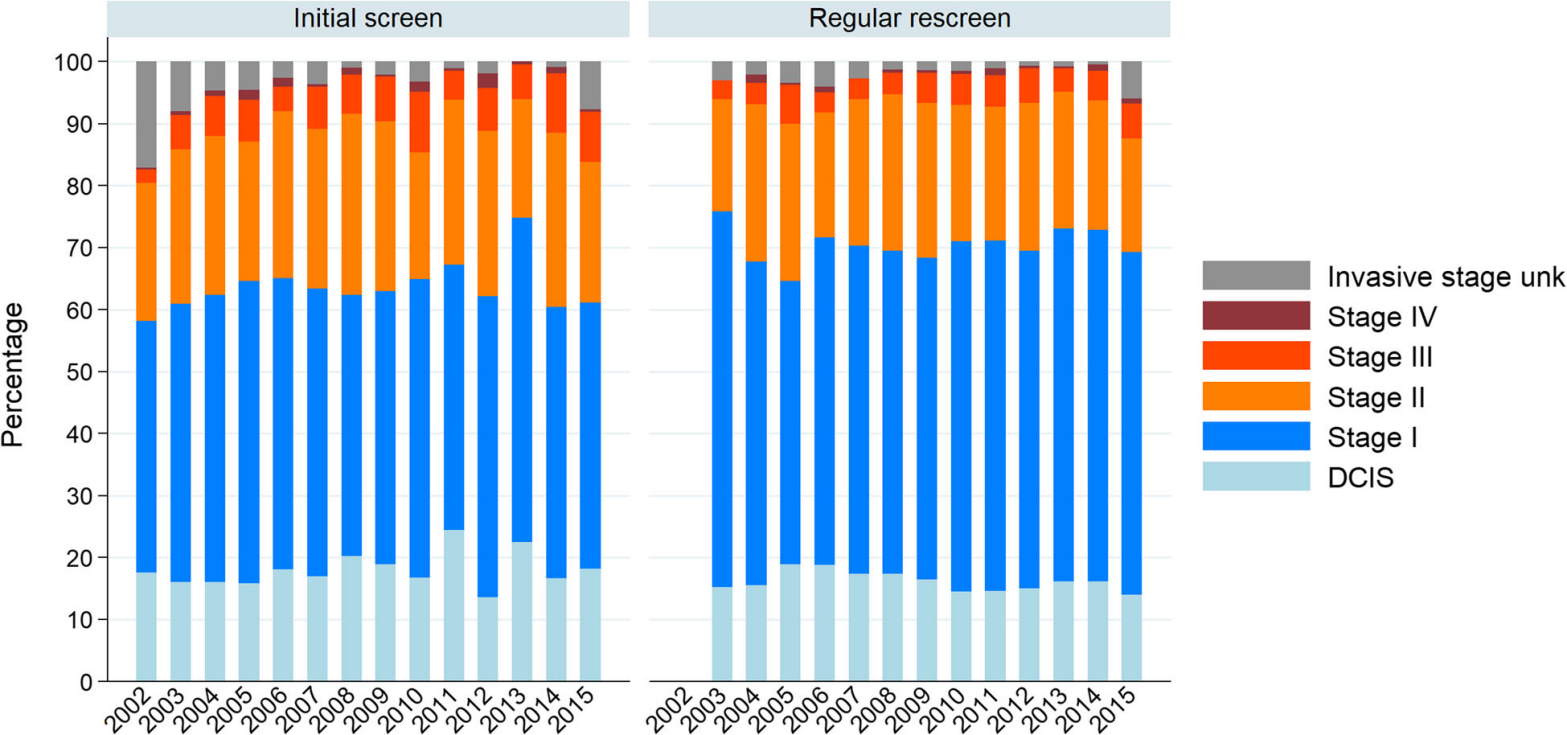
Stage distribution among all screen-detected cancers. Analysed by screening round, Flanders Belgium 2002–2016

Figure 3 and Table 4 show the distribution of tumor stage. There appears little difference between the distribution of initial and subsequent screens, which is surprising.

The proportion of DCIS was 18.2 and 14.0% in 2015 (initial and subsequent regular screens respectively), without a significant trend.

The proportion of tumours stage II+ was 31.2 and 24.8% in 2015 (initial and subsequent regular screens respectively). There is a significant trend only in the initial screens (AAPC + 1.9%).

Benchmark targets for DCIS distribution were achieved. The benchmark for stage II+ were not achieved in initial screenings, while 2015 was the first year they were achieved for subsequent regular screens.

### Nodal status of screen-detected cancers

**Fig. 4:**
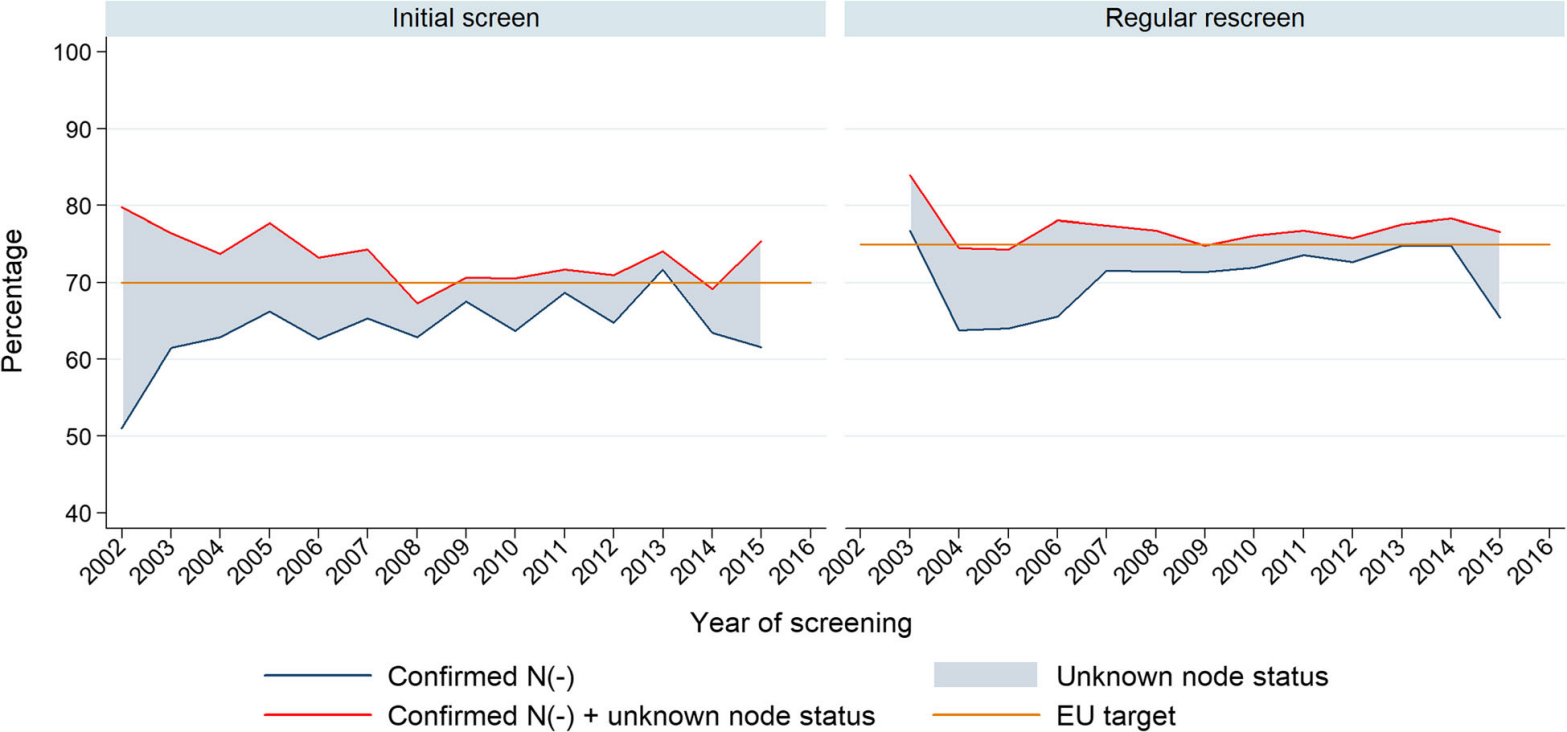
Node status distribution among all invasive screen-detected cancers. Analysed by screening round, Flanders Belgium 2002–2016

The proportion of node negative cases among all invasive SDC was 61.6 and 65.4% in 2015 (initial and subsequent regular screens, respectively), without a significant trend (Fig. 4 and Table 4). This is below EU targets. The proportion of invasive SDC for which nodal status was unknown was 7.7 and 11.2% in 2015 (initial and subsequent regular screens respectively). Figure 4 also shows what the proportion of node negative SDC would be if all these unknown cases turn out to be node negative.

## Discussion

We analysed key performance indicators for the Flemish PMSP for the period 2002–2016.

A much larger fraction of the population was covered in 2016 (64.2%) compared to the start of the programme (46.2% in 2003), even though the response to the screening invitation remained stable throughout 15 years. The growth in coverage slowed down after the majority of women started receiving timely invitations (93.2% in 2011). This indicates that the PMSP coverage increase was not so much the result of a change in intention to screen among the target group, but was instead largely due to the fact that more women were receiving their invitation on time.

Opportunistic screening was well established in Belgium long before the PMSP started [12]. Between 2003 and 2016, opportunistic coverage gradually decreased (AAPC −3.0%). Many of these women gradually switched to the PMSP. Several factors may have encouraged this switch: the quality of the opportunistic screening is not guaranteed (quality assurance of equipment, double-reading, etc.), opportunistic screening is not entirely free of charge, and booking appointments for a PMSP screening requires less effort from the women. Nevertheless, 14.1% of women preferred opportunistic screening in 2016. Previous studies indicate that physician’s advice is the primary reason for not switching [13].

The decrease in recall rate, combined with the stable CDR, means that fewer women are receiving a false-positive recall (20.2/1000 screens in 2016) leading to a higher positive predictive value of the screening mammograms (21.3% in 2016), which is also above the EU mean of 12.2% [3]. There are several hypotheses for this. Firstly, yearly symposia on lowering recall rate have been organized by the CvKO since 2010. Secondly, individual 4-monthly feedback is sent to all readers since 2008–2009. These reports compare their individual recall rate with the anonymised rates of their colleagues. Thirdly, the introduction of digital mammography screening, which led to an increased CDR in other countries [14], occurred in the same period as the reduction of the recall rate. Theoretically, the introduction of digital screening could have increased the CDR and thereby masked the lowering of CDR due to more restrictive recall strategy. However, this is unlikely as previous research has shown that digitalization in Flanders did not result in significantly different cancer detection rates [15]. Although the lowering of recall rate in combination with a stable CDR is a positive evolution, it is necessary to evaluate the negative counterpart i.e. interval cancer rate. More specifically, a review of interval cancers could determine whether breast cancers are more likely to be missed compared to other countries.

Surprisingly, the tumour stage distributions hardly differ between initial and subsequent regular screening. The same is true for CDR: in 2016 the CDR was 5.0‰ in subsequent regular screens (EU mean 5.6‰) and 6.3‰ in initial screens (EU mean 7.2‰) [3]. This could be explained by a large proportion of “initial screens” which were preceded by opportunistic screening [16]. In 2019, the CvKO will pilot a method that adjusts the KPIs of initial screens for the occurrence of such preceding opportunistic screening.

Benchmark targets for nodal status have not been achieved in 2015. This could be partly caused by the fact that more than 10% of 2015’s invasive SDC still have unknown nodal status. Assuming at least some of these unknown cases are node negative, the benchmark targets might be achieved.

Programme sensitivity is stable (65.1% in 2014) but lower than in other countries such as Germany (78.2%) [17], the Netherlands (74.4%) [18], Norway (75.5%) [19], or Canada (68%) [20]. Closer inspection reveals that the categorization of BC as either SDC or interval cancers differs between programmes. For instance, in the German programme any BC found within 24 months after a positive screening was considered an SDC, while the Canadian Programme only considered a BC as screen detected if they were found within 6 months after a positive screening [17, 20]. The Canadian programme will thus classify certain BC as interval cancers, while the German programme would see them as SDC. Such differences will influence programme sensitivity. The Flemish PMSP only considers a BC as screen detected if it was found within 3 months after the first diagnostic assessment that follows a positive screening (see also Table 2) [21]. The Canadian definition of an SDC is relatively close to the Flemish, which might explain why their programme sensitivity is similar (68% in Canada, 65.1% in Flanders) [20].

To decrease the risk of missing BC (thereby increasing sensitivity), the CvKO started a self-teaching project in 2018 which provides all readers with a yearly list of BC for which they had made a negative reading. To counter a possible increase in recall rate, readers also receive a list of their positive readings in which no breast cancer was found in the 2 years following screening.

The major strength of this first nationwide analysis of KPIs in the Flemish PMSP is the availability of national data on all mammographic PMSP screenings performed over 15 years, together with the matched oncological follow-up data from the BCR. The completeness of BCR breast cancer data was previously estimated to be 99.7% [6].

Our study also has some limitations. Firstly, not all screened women provided an informed consent to link their screening data to the BCR data, mostly during the programme initiation in 2002 and 2003. Refusal rates fluctuated around 1% or less of screened women. Secondly, we suspect that some of the “initial screens” in the programme are in fact preceded by opportunistic screen. We are investigating this further. Thirdly, some of the tumor characteristics have missing data, meaning the proportions calculated for those KPIs might still rise. For instance, in 2015 65.4% of invasive BC were node-negative, but a further 11.2% had unknown nodal status. The same is true for stage distribution. Fourthly, we considered all diagnostic mammograms as opportunistic screening, even though a minority are undoubtedly for diagnostic purposes [12]. The BCR and CvKO are currently investigating the proportion of all diagnostic mammograms that are for screening purposes. Fifthly, in the current analysis, we cannot estimate the impact on breast cancer mortality. The CvKO participates in the EU-topia project (https://eu-topia.org) to attempt to obtain an estimate, while the BCR is currently performing its own analysis.

## Conclusion

Besides the suboptimal attendance rate, most performance indicators in the Flemish PMSP meet EU benchmark targets. Nonetheless, there are several priorities for further investigation. Firstly, the response to invitation has remained stable, indicating that the strategies that have been used to increase screening uptake these last 15 years have had limited effect. Now that the invitation scheme has been optimised, a critical evaluation should be made of these strategies. Secondly, interval cancers should be analysed by individual radiological review as described in the European guidelines [4]. If the proportion of “missed cancers” is comparable to the results in other countries, it can be concluded that Flanders has found a successful way of reducing recall rate while maintaining a stable CDR. The ensuing lower number of false positive screenings will lead to increased rescreening rates [22, 23]. Thirdly, ways must be found to further limit the occurrence of interval cancers after positive screenings with negative diagnostic assessment. One possibility could be to let diagnostic assessment only take place in specialised centres. Fourthly, the clinical and health economic impact of the PMSP should be analysed, along with the effect of opportunistic screening on CDR in initial and subsequent irregular screens. The BCR and CvKO are therefore analysing the impact of mammographic screening in three scenarios: women attending PMSP, women attending only opportunistic screening, women attending both screening types. Among other things, the study will compare cost-effectiveness and clinical outcome. This is being done in parallel with efforts to estimate the impact on breast cancer mortality.

## Data Availability

The datasets used and analysed during the current study are closed to public access, but access can be requested by contacting the corresponding author or on www.bevolkingsonderzoek.be.

## Abbreviations

AAPC: Average Annual Percentage Change
APC: Annual Percentage Change
BC: Breast cancer
BCR: Belgian Cancer Registry
CDR: Cancer detection rate
CvKO: Centre for Cancer Detection
DCIS: Ductal carcinoma in situ
DR: Direct radiography
KPI: Key performance indicator
PMSP: Population-based mammography screening programme
PPV: Positive predictive value
SDC: Screen-detected cancer
